# Risk factor for mortality associated with carbapenem-resistant Enterobacteriacea infections

**DOI:** 10.1186/cc12932

**Published:** 2013-11-05

**Authors:** Cláudia MDM Carrilho, Cintia MC Grion, Marcos T Tanita, Jamile Vale, Larissa Oliveira, Ana P Marchi, Silvia F Costa

**Affiliations:** 1 Universidade Estadual de Londrina, PR, Brazil; 2 Universidade de São Paulo, SP, Brazil

## Background

Antimicrobial resistance has emerged and increased in the last 25 years, complicating the treatment of nosocomial infections, especially for extended-spectrum beta-lactamase Enterobacteriacea (ESBL). Furthermore, disseminated use of invasive procedures, particularly in ICU patients favors the emergence of multiresistant pathogens. More recently, Enterobacteriacea has developed a new mechanism of antimicrobial resistance and became resistant to carbapenems. Among Enterobacteriacea resistant to carbapenem, *Klebsiella pneumoniae *is the most common. These pathogens manifest resistance to most of antimicrobials tested and are associated with high mortality rates. The aim of this study is to describe epidemiologic data about nosocomial infections due to carbapenem-resistant Enterobacteriacea and identify risk factors for death.

## Materials and methods

Longitudinal study evaluating patients with infections caused by carbapenem-resistant Enterobacteriaceae, isolated from blood, urine, tracheal secretions, skin and soft tissues, treated accordingly to the Brazilian Society of Infectious Diseases guidelines, from March 2011 to December 2012. Acute Physiology and Chronic Health Evaluation (APACHE II) was calculated to evaluate severity of disease and Sequential Organ Dysfunction Assessment (SOFA) to measure organ dysfunction. Comorbidities were classified according to Charlson comorbidities index list. Patients were followed until hospital discharge.

## Results

During the study period, 174 nosocomial infections caused by carbapenem-resistant Enterobacteriaceae were identified in 148 patients. All infections were microbiologically documented and 136 (78.2%) occurred in patients who were admitted to the ICU. Sepsis (17.8%), polytrauma (14.4%), cardiovascular disease (13.8%) and respiratory disease (11.5%) were the most common diagnosis. Most of the patients (78%) had one or more comorbidities according to Charlson criteria, and 57/148 (43.2%) patients had three or more comorbidities. Median APACHE II was 20.7 (7 to 38) and median SOFA at ICU admission was 7 (0 to 14). Median length of hospital stay was 43 (6 to 230 days). *K. pneumoniae *was the most common enterobacteria (86.8%), followed by Enterobacter spp. (8%). The mechanism of resistance was identified as Klebsiella pneumonia carbapenemase (KPC) present in 77.2% of *K. pneumoniae *infections. Shock was present in 81/148 (46.6%) patients and dialysis was used in 62/148 (35.6%). Hospital mortality was 62.6% and associated mortality was 33.3%. Multivariate analysis identified dialysis and pneumonia as independent risk factors for death.

## Conclusions

Many patients infected with carbapenem-resistant Enterobacteriae were identified, and most of them were caused by carbapenemase producing bacteria. These infections were associated with high mortality rate. Shock, dialysis, pneumonia, SOFA discharged >6 and APACHE >20 were identified as independent risk factors for mortality.

